# Wake-up call for HPPP – health promotion, prevention, and preparedness

**DOI:** 10.3389/fpubh.2023.1249408

**Published:** 2023-10-05

**Authors:** Vered Lev, Aviva Ron

**Affiliations:** ^1^Stanford University School of Medicine, Stanford, CA, United States; ^2^Independent Consultant, Rosh Pina, Israel

**Keywords:** public health, health promotion, prevention, health promoting settings, health policy

## Abstract

The latest public health emergencies exposed urgent gaps in health promotion, prevention and preparedness (HPPP). Existing and new infectious diseases have gained far more prevalence than expected, and inequities in access to health care accounted for disturbing differences in the toll of these diseases in different populations. The COVID-19 pandemic not only demonstrated the need to prevent the onset and progression of non-communicable chronic diseases (NCDs) and promote healthy lifestyles, but also the need to prepare for new infectious diseases and their long-term effects on both physical and mental health. Preparedness was previously associated with natural disasters, with activities directed to developing emergency humanitarian action response resources. However, these actions are inadequate for the frequent natural disasters as the climate crisis intensifies. To reach effective actions in HPPP, we take a broad approach to HPPP components, identify the main stakeholders and suggest methods to change allocations for HPPP. We propose a call for action at global and national levels, involving strengthening the United Nations’ Sustainable Development Goals and government commitment to HPPP.

## Introduction

## Health promotion, prevention, and preparedness

The latest public health crisis presented a wake-up call for enhanced synergies across HPPP functions, and increased collaboration between major HPPP stakeholders. Health promotion aims to encourage healthy personal lifestyles as well as public environmental conditions to make this possible. Prevention is more complex as it generally includes three parts: primary prevention to prevent the onset or transmission of disease, secondary prevention to prevent severity after the onset of a disease, as well as complications of conditions, and tertiary prevention to prevent disability resulting from a disease or injury. These functions are the foundation for reducing the transmission of diseases and the onset and increase in severity of many chronic conditions. Preparedness is a set of activities, from recognition of the potential hazards and the preparation of resources to deal with epidemics, as well as emergency humanitarian action when needed. We have taken a broad approach to HPPP as effective implementation requires understanding of each component, their dependencies, and the role of the partners and stakeholders.

## Wake up call for HPPP actions

### The COVID-19 pandemic and the soaring rates of NCDs

The COVID-19 pandemic demonstrated that the spread of infectious diseases is still an enormous public health hazard. Older people were disproportionately impacted by the COVID-19 pandemic; in the US, adults aged 65 and over comprised about 80% of total COVID-19 deaths in 2020 (1). While older adults were labeled as the most vulnerable and urged to stay at home, we did not consider the known negative health outcomes of social isolation, as higher risk of dementia, coronary artery disease, and mortality (2). We failed to predict the negative impact of COVID-19 social isolation on mental health (3). In the working-age population, isolation and the loss of income led to increased domestic violence, substance abuse and depression (4, 5).

We see the urgency of preventing the spread of old and new infectious diseases and a continued need to promote healthy lifestyles and prevent the onset and progression of NCDs. We now recognize the negative interaction between the two groups; as reflected in more deaths and residual morbidity in people with risk factors such as obesity and multiple chronic conditions who were infected with different variants of COVID-19 (6, 7). While there are continuous efforts to prevent the onset and progression of NCDs, they are still considered an emerging global health threat, killing 41 million people annually (8). The pandemic has shown the need for preparedness, both for long-term care of people with NCDs and infectious diseases with long-term effects. We have been warned about future pandemics and should not repeat mistakes. Preparedness is not a concept for the future: it is required immediately to deal with the health care needs of the consequences of the COVID-19 pandemic and expectations of future pandemics.

### Increasing natural disasters linked to climate change

Preparedness was previously associated with natural disasters, with activities directed to developing emergency humanitarian action response resources. First, through the establishment and reinforcement of United Nation’s (UN) organizations, as the UN Disaster Assessment and Coordination Organization (UNDAC) which is part of the international emergency response system for sudden-onset emergencies, and the Office for the Coordination of Humanitarian Aid (OCHA), as well as the specialized agencies such as the World Health Organization (WHO), and the Red Cross. These agencies provide the operational guidelines for non-government disaster organizations, thereby coordinating all humanitarian emergency action at the time of the disaster and after. For example, as part of WHO’s disaster preparedness and response efforts, the agency formed the *Disaster Management Guidelines: Emergency Surgical Care in Disaster Situation*. The comprehensive manual provides areas affected by natural disasters with guidance on the management of common injuries encountered in disaster situations (9).

However, these actions are inadequate for the more frequent natural disasters now linked to climate change. We see an unprecedented number of natural disasters with more displaced persons, and outbreaks of diseases associated with unsafe water and sanitation, such as diarrheal diseases which are the second leading cause of death in children under five worldwide (10). As climate change intensifies, wildfires are also on the surge, with a global increase in extreme fires of up to 14 percent by 2030 and 30 percent by the end of 2050 (11), causing increased pollution in large cities with preexisting major pollution issues and long-term adverse health consequences, especially among vulnerable populations (12).

## Reaching effective HPPP measures

The first step for effective HPPP actions is to identify the factors, actors and methods to reach the HPPP goals, based on the evidence to date. For example, HPPP measures in maternity care are showing astonishing outcomes. The Maternal Mortality Ratio (MMR, maternal deaths per 100,000 live births) is a health indicator with one of the biggest variations, with a range of 3 in Finland and Norway, 19 in the USA, to a reported 1,150 in South Sudan and estimated 1,800 in Afghanistan (13, 14). The huge variation shows a clear example of how appropriate HPPP measures through health promotion as healthy nutrition, using appropriate vaccinations and scans to prevent and preparedness actions as can prevent morbidity and mortality in both mothers and infants.

To reach effective HPPP, the questions that need to be asked are who are the stakeholders, how to engage them, strengthen the partnerships, and allocate resources to these functions in the framework of a global commitment.

### We identify the main stakeholders as

***Governments***, committed to reaching the Sustainable Development Goals (SDGs), are major stakeholders. Yet government programs tend to be vertical, with limited funding channeled through separate ministries. The Health in All Policies (HiAP) approach introduced a collaborative effort that integrates and articulates health considerations into policymaking across sectors to improve the health of all communities and people (15, 16). The mechanisms of HiAP are complex and have been found to work well in some local government contexts but less so at national efforts to enhance HPPP. Governments still need to be convinced of the effects and value of HiAP in progressing a cross-agency health promotion agenda. Governments that do use HiAP face the challenges in changing legislation and structures to facilitate policy coordination (17). The responsibility for HPPP indeed has to be collaborated between all ministries. In reality, implementation is generally delegated to local government until an emergency occurs, such as a cholera outbreak, and only then we may see collaboration between local authorities. Enforcement of regulations may be beyond the capacity of local authorities, with their own internal political pressures on licensing and inspection, as banning the sale of cigarettes and alcohol to minors. These harsh realities include the fact that many local authorities are underfunded and mismanaged.

The worst scenario is when the major source of financing for health care is out-of-pocket payments by patients. Governments in these countries may take responsibility for immunization against communicable childhood diseases but do not invest enough resources and money on personal preventive care. Low-and mid-income countries are also less likely to spend funds on preparedness, despite the fact that housing, roads and bridges are often substandard and do not sustain the damage of natural disasters. Infectious diseases are far more transmissible in substandard housing with poor ventilation and inadequate water and sanitation facilities (18).

Improved legislation and regulation are urgent. Not all governments have the knowledge and tools to draft legislation and regulations on HPPP, allocate funds or find new fiscal space for the measures required. This is where the specialized UN organizations come in, through their resolutions, conventions and guidelines on monitoring indicators, such as in the SDGs. These organizations have the mandate to develop tools which can be followed by all countries – a fact that should not be ignored. The dissemination of these standards can also guide the multi-and bilateral donors in development aid in the framework of an informed policy backed by legislation.

***The United Nations*** representing the collective body of member states, has developed tools to motivate governments to improve the quality of life of their citizens. The Millennium Development Goals (MDGs) and now the SDGs for 2030 serve as goals with explicit targets (19). These tools can guide the allocation of resources to achieve the goals.

Within the UN specialized organizations, the ***World Health Organization*** is charged with the development of public health provision through health promotion guidelines. WHO’s work in the past was mainly directed at Ministries of Health. The UN undertakings of reaching the MDGs and now SDGs have led to some but not enough shift from vertical programs to a cross-cutting but not adequately coordinated approach.

The next UN body as a stakeholder is the ***International Labour Organization (ILO)***, which has dealt with health promotion and prevention since its founding in 1919, through its conventions and standards. The major areas of these conventions with specific prevention approaches are maternity protection, health care under the social security framework, occupational safety and child labour. *Decent Work* is a later initiative of the ILO introduced in 1999, calling for creating jobs, guaranteeing rights at work, extending social protection, and promoting social dialogue (20). These four pillars of the *Decent Work* agenda became integral elements of the 2030 agenda for SDGs as Goal number 8 (19).

***Social Security schemes*** are major stakeholders and probably have the most to gain from effective HPPP. But concern with their financial viability has grown as a result of some stagnation in economic growth and an increasing informal sector economy globally. Promotion and prevention are not adequately considered in cost control efforts of the schemes and there is little consideration of expenditure in one branch, such as health care, as savings in another branch, such as disability. The transfer of funds between schemes providing short and long-term payments could be a serious driver in our efforts to increase funds for HPPP23. For example, targeted health promotion on lifestyle, physical activity and nutrition may lead to healthier adults in working age needing less cash sickness benefits during sickness absenteeism.

To put it simply, we have a positive interaction of expenditure and savings between life contingencies. The expenditures on these contingencies are usually funded by separate contributions or sources and may be managed by separate organizations. While the transfer of funds from one benefit branch or scheme to another may not currently be permitted by law, ways for collaboration and cooperation between the different partners, when the cost benefits are clear, and the outcome is a healthier and more productive population could be explored.

***Multi and bi-lateral funding agencies*** have been major players through grants and loans for diseases with the highest risks of spread. The recipient countries have not always had the capacity to use the funds in an optimal and sustainable way. When the aid programs were successful, they have often been perceived as being good for the lowest-income populations, without due consideration of how these actions could benefit populations in other countries, both high and low-income (21).

There are additional stakeholders, such as private corporations and foundations, the academic community, non-government organizations dealing with specific diseases and patient associations. Their role may vary in different countries, but they should be considered in national plans.

## Conclusion and recommendations

There is no “go it alone” in HPPP. At a global level, we propose that the UN broaden the scope of the SDGs, just as *Decent Work* was upgraded to a priority area in SDG 8. The areas covered here can be included in other SDGs. All the relevant specialized UN agencies need to be part of the effort to provide the impetus to countries to get back on track in reaching the SDGs after the delays noted at the United National General Assembly (UNGA) in September 2022 (22).

At the national level, governments have to commit to an ideology that leads to policy and legislation on appropriate HPPP.

### The conditions for successful HPPP need to include

- Continued identification of effectiveness in HPPP measures. The promotion of routine tests for early detection of specific malignancies and relevant vaccination, such as the Human Papillomavirus vaccines against cervical cancer shown to be an effective HPPP measure that needs to be amplified in other areas as well.
- Adoption of mechanisms to recognize how expenditure by one authority or agency can impact on savings in other sectors, with collaboration between agencies to pool funds for HPPP.
- Recruitment of professionals outside the health field to enhance rational and logical thinking and undertake operations to increase responsibility and accountability in public health systems.
- Improved decision-making mechanisms, with input from successful private corporations and through the enlistment of trusted leaders from the cultural environment of the population, using appropriate communications mechanisms.
- Measures to link research findings to legislators and providers of health care.
- Advancements in the input from health professionals by the implementation of changes in basic as well as continued education to improve knowledge and counseling on risk factors, such as food security and ecology.
- Involvement of relevant communications systems and social media in implementing the HPPP policies.
- The ability to evaluate and change policies and legislation in a timely manner to meet the new needs of changes in the disease pattern and demographic transitions.

As the timeline for the Sustainable Development Goals for 2030 is fast approaching enhanced collective efforts are crucial. Similarly to the Health in All Policies approach, success requires interaction between different sectors. The last of the SDGs is *Build Partnerships for the Goals*, and this is perhaps the greatest challenge. It requires giving up exclusiveness and sharing credits in a way that has not been done before for the good of all. Public health is about the good of all for a sustainable future.

## Data availability statement

The original contributions presented in the study are included in the article/supplementary material, further inquiries can be directed to the corresponding author.

## Author contributions

VL and AR contributed to the conception and design of the paper and wrote the first draft of the manuscript. AR organized the structure of the paper and did the first edit the paper. VL did the second edit of the paper. All authors contributed to the article and approved the submitted version.
